# Mesenchymal Phenotype Predisposes Lung Cancer Cells to Impaired Proliferation and Redox Stress in Response to Glutaminase Inhibition

**DOI:** 10.1371/journal.pone.0115144

**Published:** 2014-12-12

**Authors:** Danielle B. Ulanet, Kiley Couto, Abhishek Jha, Sung Choe, Amanda Wang, Hin-Koon Woo, Mya Steadman, Byron DeLaBarre, Stefan Gross, Edward Driggers, Marion Dorsch, Jonathan B. Hurov

**Affiliations:** Agios Pharmaceuticals, Cambridge, Massachusetts, United States of America; University of Parma, Italy

## Abstract

Recent work has highlighted glutaminase (GLS) as a key player in cancer cell metabolism, providing glutamine-derived carbon and nitrogen to pathways that support proliferation. There is significant interest in targeting GLS for cancer therapy, although the gene is not known to be mutated or amplified in tumors. As a result, identification of tractable markers that predict GLS dependence is needed for translation of GLS inhibitors to the clinic. Herein we validate a small molecule inhibitor of GLS and show that non-small cell lung cancer cells marked by low E-cadherin and high vimentin expression, hallmarks of a mesenchymal phenotype, are particularly sensitive to inhibition of the enzyme. Furthermore, lung cancer cells induced to undergo epithelial to mesenchymal transition (EMT) acquire sensitivity to the GLS inhibitor. Metabolic studies suggest that the mesenchymal cells have a reduced capacity for oxidative phosphorylation and increased susceptibility to oxidative stress, rendering them unable to cope with the perturbations induced by GLS inhibition. These findings elucidate selective metabolic dependencies of mesenchymal lung cancer cells and suggest novel pathways as potential targets in this aggressive cancer type.

## Introduction

It has been appreciated for 60 years that glutamine (Gln) is a conditionally essential amino acid for the growth of cancer cells in culture [1]. Glutamine is the most abundant circulating amino acid in humans at a concentration of ∼500 µM in serum, and many studies indicate that Gln is a major source of carbon and nitrogen for tumor cells [2], [3]. One enzyme in particular, glutaminase, localizes to the mitochondria and appears to be critical for entry of glutamine carbon into the tricarboxylic acid (TCA) cycle in many cancer cells [4], [5]. Glutamine is converted to glutamate and ammonia by glutaminase, and the glutamate carbon is subsequently shuttled into the TCA cycle via conversion to α-ketoglutarate (α-KG) by several different enzymes including glutamate dehydrogenase and various aminotransferases. Mammals carry two genes that encode mitochondrial glutaminase, *GLS1* (or KGA) and *GLS2* (or LGA), which were initially identified in normal kidney and liver, respectively [6]. The *GLS1* gene produces two predominant splice variants encoding canonical GLS1 (also known as KGA) and GAC, however the differential functions of these two variants are not well understood [7].

Several elegant studies have illustrated the contribution of carbon derived from Gln into the TCA cycle via glutaminolysis [8], [9]. *GLS1* expression has also been identified as a target of the myc oncogene [10] and has been implicated as an effector of Rho-mediated transformation in breast cancer cell lines [11]. Interference with GLS activity via either genetic or pharmacologic manipulation has been demonstrated by a number of groups to negatively impact the growth of select cancer cell lines [11]–[13]. Based on the totality of published data on the importance of Gln and glutaminase in cancer, GLS has been highlighted as a potential drug target for oncology indications [14]. To our knowledge *GLS1* is not mutated or amplified in human cancers. In order to facilitate the use of GLS1 inhibitors in the clinic a better understanding of the genetic and phenotypic contexts that drive dependence on GLS1 is required.

In this study we validate the on-target cell-based activity of a published GLS1 inhibitor, BPTES (bis-2-(5-phenylacetamido-1,2,4-thiadiazol-2-yl)ethyl sulfide) [15], showing that it acts specifically via a GLS1-dependent mechanism to induce anti-proliferative and metabolic perturbations in cells. We use BPTES as a validated tool compound to screen a panel of lung cancer lines and identify a subset of lines that exhibit GLS dependence and express markers characteristic of a mesenchymal phenotype. TGF-β mediated induction of EMT sensitized cells to GLS inhibition and was associated with impaired mitochondrial respiratory capacity and increased sensitivity to oxidative stress. These findings point to a selective role for GLS in mesenchymal NSCLC cells, which utilize GLS to provide a carbon source for oxidative phosphorylation and to maintain redox balance required for cellular proliferation.

## Results

### Cell line panel screen and validation of BPTES as a tool compound

**Figure 1:**
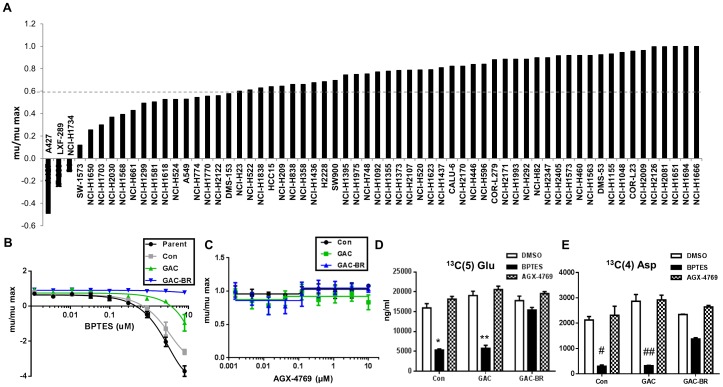
Cell line panel screen and validation of BPTES as an on-target tool compound. (A) Sensitivity of a panel of NSCLC lines to inhibition of growth by 10 µM BPTES in a 72 hr proliferation assay. Growth rates (mu) plotted relative to DMSO control for each cell line (mu/max). (B,C) A427 parent cells or cells stably expressing an empty vector control (Con), wild-type GAC (GAC), or a BPTES-resistant GAC enzyme (GAC-BR) were treated with the indicated concentrations of BPTES (A) or the inactive BPTES analogue, AGX-4769 (C), in a 72 hr proliferation assay. Results are representative of three independent experiments with mean and standard deviation indicated. (D,E) Measurement of isotopomer labeled ^13^C(5)-Glu (D) or ^13^C(4)-Asp (E) from cells treated for 4 hr with BPTES or with inactive analogue AGX-4769. Results are the mean of three replicates with the standard deviation indicated. Calculated p-values from student t-test, ^*^(3×10^−8^), ^**^(10^−9^), ^#^(10^−7^), ^##^(2×10^−6^).

To gain insight into the genetic/phenotypic determinants of GLS dependence, we screened a panel of cell lines with the published GLS1 inhibitor BPTES [15]. We focused specifically on one tumor type, lung cancer, due to the large number of established cell line models available. Of the 62 lung lines selected for analysis using BPTES, 44 have been classified as NSCLC (S1 Table in S2 File). Relative growth rates (mu__BPTES_/mu__DMSO_) were calculated following drug treatment in order to best compare relative sensitivity to BPTES between cell lines that varied in doubling time. Approximately 30% of these 62 lines exhibited significant sensitivity to BPTES as defined by>40% reduction in growth rate (Fig. 1A, S1 Table in S2 File). The degree of sensitivity to BPTES was highly varied, with cell death evident in some cell lines (µ_BPTES_/µ_DMSO_ <0; e.g. A427) and a near complete lack of response in others (e.g. NCI-H1563). Since CellTiter-Glo (CTG), which measures ATP levels, was used as a rapid, surrogate method for screening cell number following BPTES treatment, we validated that the effects of glutaminase inhibition on cellular ATP levels was a representative readout of cell number. Assessment of cell number by DNA content (Syto60) demonstrated a comparable effect of BPTES treatment on cell proliferation as indicated by CTG at the 72 hr endpoint (S1 Figure in S1 File).

In order to confirm that the effects of BPTES on cells are on-target, we engineered A427 cells to overexpress cDNA encoding a previously described BPTES resistant mutated GAC, named GAC-BR (F318A and F322A) and assessed the effect of BPTES on cell growth of these cells compared to cells overexpressing wild-type GAC (GAC-WT) or an empty vector. GAC-BR is unable to bind BPTES but maintains catalytic activity [16]. Overexpression of GAC-BR rendered the A427 cells resistant to the growth inhibitory effects of BPTES (Fig. 1B) whereas cells expressing empty vector or GAC-WT retained significant sensitivity to BPTES. The modestly blunted effect of BPTES on growth of the GAC-WT compared to the parental or empty vector expressing lines is likely due to the ∼2x increase in GAC expression in this model. We also utilized this system to address the on-target effects of BPTES in modulating de novo synthesis of glutamate and aspartate from glutamine. S2 Figure (in S1 File) highlights the flow of carbon from glutamine to glutamate and downstream TCA intermediates as has been described [8], [9]. Overexpression of GAC-BR, but not GAC-WT, ameliorated the BPTES induced block in glutamine flow into glutamate and aspartate (Fig. 1D,E). The incomplete restoration of ^13^C(5)-aspartate levels may reflect the particular sensitivity of this metabolite to GLS inhibition, perhaps due to the contribution of both carbon and nitrogen from glutamine to aspartate biosynthesis and/or due to effects from inhibition of the endogenous GLS1 activity. A biochemically inactive BPTES analogue, AGX-4769, with a conservative modification (S3 Figure in S1 File), was used to treat the same cell lines and had no effects on proliferation or downstream metabolites (Figs. 1C-E). These data indicate that the effects of BPTES on proliferation and modulation of downstream metabolites is due to inhibition of GLS1 and not due to off-target effects.

As yet another means of validating the utility of BPTES as a tool compound to screen for GLS dependence, we performed siRNA mediated knockdown of GLS1 (either total or GAC specifically) in a subset of 6 of the NSCLC lines from the panel. All 3 lines that were sensitive to BPTES were also sensitive to GLS KD, while the 3 that were not sensitive to BPTES were less/not sensitive (S4 Figure in S1 File). While KD of GAC alone resulted in substantial growth inhibition in the BPTES sensitive lines, KD of total GLS1 resulted in an even more profound impairment indicating that both isoforms of GLS1 (KGA and GAC) contribute to growth/viability of these cells.

### GLS1 dependence in NSCLC with a mesenchymal (EMT) phenotype

**Figure 2:**
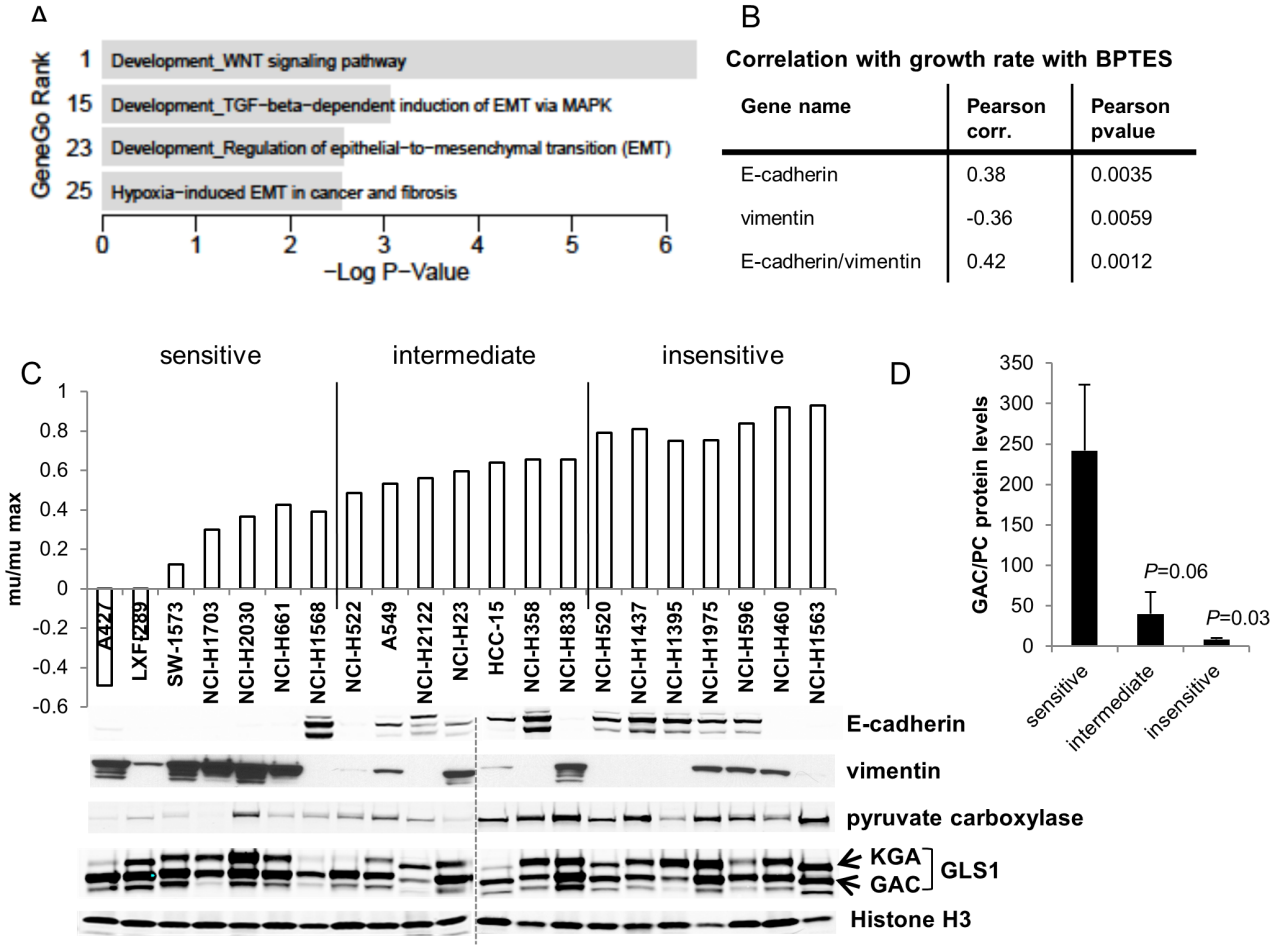
Association between EMT-related markers and BPTES sensitivity in NSCLC lines. (A) GeneGo analysis indicating four top-ranking mesenchymal signatures correlating with BPTES sensitivity. P-values are calculated within the GeneGo software using the hypergeometric distribution. (B) Anti-correlation between high E-cadherin low vimentin RNA expression levels and sensitivity to BPTES. (C) Mu/mumax values depicted following 72 hr treatment of NSCLC lines with 10 µM BPTES. Equal protein amounts of tumor cell line extracts were electrophoresed, with the group of more sensitive lines on the left and least sensitive on the right, and immunoblotted for the indicated proteins. Histone H3 was used as a loading control. (D) Quantitation of average GAC protein levels relative to PC protein expression (+/−SEM) in BPTES sensitive (n = 7), intermediate (n = 7), and insensitive (n = 7) lines. *P* = 0.03 comparing GAC/PC protein levels in sensitive compared to insensitive lines; *P* = 0.06 comparing sensitive to intermediate groups (unpaired 2-tail t-test).

Based on the range of BPTES sensitivity observed across the 62 cell lines (Fig. 1), we searched for transcriptional markers that associated with sensitivity or resistance using a publicly available dataset from the Broad Institute (https://array.nci.nih.gov/caarray/project/golub-00327). Of note, neither GLS1 nor GLS2 mRNA expression levels correlated with sensitivity. The top 200 probes showing most significant correlation with sensitivity were then submitted to GeneGO pathway analysis (http://portal.genego.com/cgi/data_manager.cgi). We noted that no metabolism-related pathways were strongly associated with BPTES responsiveness, indicating that any intrinsic differences in metabolism between sensitive and resistant cells are not broadly reflected at the transcriptional level (Table S2 in File_S2). Interestingly, 4 of the top 25 pathways were related to EMT (ranks 1, 15, 23, 25; Fig. 2A). Immune response pathways were also highly represented in this analysis. While we chose to focus our investigations on the link between EMT and GLS1 dependence, it is notable that a link between NF-κB and glutaminase activity has been demonstrated by others [11], [17].

In order to identify specific markers predictive of BPTES sensitivity, we probed the list of significantly correlated probes from the above analysis for individual EMT related genes. E-cadherin and vimentin were positively and negatively correlated with growth in the presence of BPTES (i.e. lack of sensitivity to GLS inhibition), respectively (Fig. 2B). Low E-cadherin/high vimentin are well-defined markers of cells that have a mesenchymal phenotype [18]. A significant correlation was also observed for several other genes typically modulated during EMT, including claudin4/7 and ZEB1 (Table S3 in File_S2). Following the initial BPTES screen, a subset of 21 of the NSCLC lines was re-examined for BPTES sensitivity and analyzed for protein expression levels of selected markers identified from the transcriptional profiling analysis (Fig. 2C,D; Table S4 in File_S2). In 6/7 of the top sensitive NSCLC lines, low E-cadherin and high vimentin protein levels were reproducibly observed (Fig. 2C). The group of sensitive lines were also marked by an average higher ratio of GAC/pyruvate carboxylase (PC) protein levels (Fig. 2C, D; *P* = 0.03). Similar results were obtained comparing total GLS1/PC levels. We chose to focus on GAC levels as this isoform predominates in most cancer cells. PC protein levels alone were significantly elevated in the insensitive compared to sensitive lines (*P* = 0.02) and mRNA levels negatively correlated with BPTES sensitivity in the larger panel of 60+ lung cancer lines screened (Pearson correlation factor: 0.29; Pearson *P* value: 0.03). Since pyruvate carboxylase can substitute for glutaminase in providing carbon to the TCA cycle [19], the association of altered GAC/PC ratios with sensitivity to GLS inhibition suggests the possibility that alternative modes of carbon entry into the TCA can influence GLS dependence.

**Figure 3:**
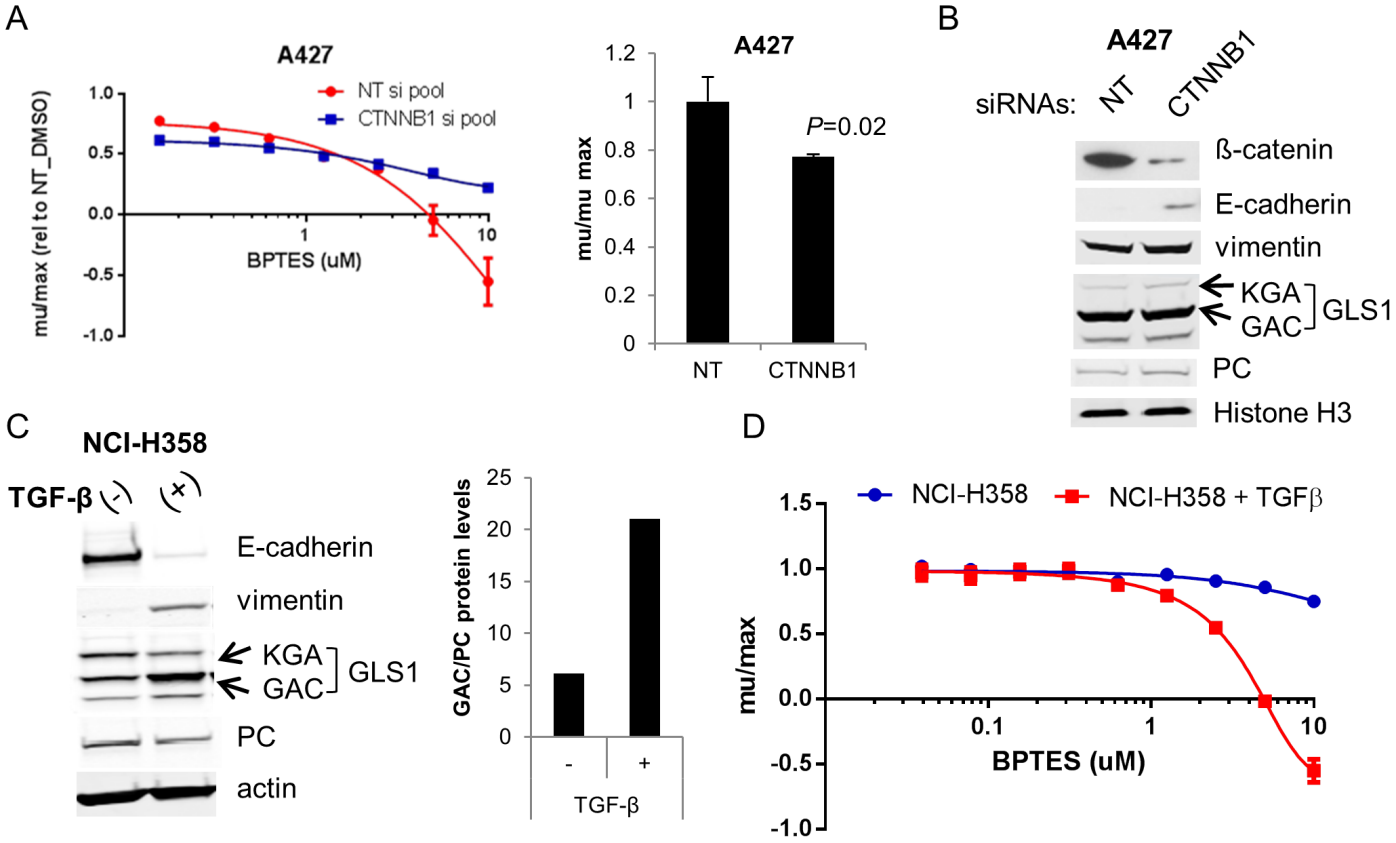
EMT drives GLS1 dependence in two independent NSCLC models. (**A**) A427 cells were transfected with non-targeting (NT) or β-catenin targeting siRNAs and treated with the indicated concentrations of BPTES (left panel). Growth rates were normalized to DMSO treated cells transfected with NT siRNAs. The right panel depicts the effects of the β-catenin siRNAs on growth of DMSO treated cells. **(B**) Immunoblot of A427 protein extracts collected from cells 72 hrs post transfection. (**C**) NCI-H358 cells were treated with 25ng/ml TGF-ß for 4 wks. Induction of EMT was confirmed by the loss of E-cadherin and gain of vimentin. EMT was accompanied by an increase in GAC relative to PC protein levels (results representative of 2 independent experiments). Actin levels indicated for protein normalization. (**D**) NCI-H358 parental and 6wk TGF-ß treated cells were treated in triplicate with BPTES for 72 hrs and cell growth assessed by CTG. Results are representative of 3 independent experiments and are plotted as average growth rates (+/−SD) compared to DMSO treated cells.

Interestingly, the three NSCLC lines that were most sensitive to BPTES (A427, LXF289, SW1573) all carry activating mutations in β-catenin (T41A in A427 and LXF289, S33F in SW1573, Table S4 in File_S2). Wnt/β-catenin signaling is one of several pathways that have been implicated in driving EMT [20]. As a result, we used siRNA mediated knockdown of β-catenin in A427 cells to determine if loss would result in a change in sensitivity to BPTES. Strikingly, β-catenin knockdown resulted in reduced sensitivity to BPTES treatment (Fig. 3A). Interestingly, the reduction in β-catenin protein levels was accompanied by an increase in E-cadherin levels (Fig. 3B), as has previously been described in bladder cancer cells [21]. In the absence of BPTES treatment, β-catenin KD resulted in a modest (23% decrease; *P* = 0.02) impairment in cell growth (Fig. 3A).

### TGFβ-Induced EMT in NCI-H358 NSCLC cells leads to GLS1 dependence

In order to better understand the contribution of mesenchymal versus epithelial phenotype to GLS dependence, we treated NCI-H358 cells, which are phenotypically epithelial (high E-cadherin/low vimentin) and exhibit moderate sensitivity to BPTES (Fig. 2C), with TGFβ to induce EMT. As has been reported [22], TGFβ shifted the NCI-H358 line to a mesenchymal phenotype as demonstrated by decreased E-cadherin and elevated vimentin (Fig. 3C). Notably, GAC protein levels were also affected during this transition, with a 2-fold increase in protein levels in the mesenchymal variant (Fig. 3C). In contrast, PC levels were slightly reduced (1.4x decrease) upon EMT, resulting in an increased GAC/PC ratio (Fig. 3C). Interestingly, concomitant with the increase in GAC expression, levels of the less prominently expressed KGA isoform decreased (∼40%) upon EMT. The regulation and potential differential function of the different GLS1 isoforms remains unclear. Consistent with the model that mesenchymal character predicts GLS1 dependence, the TGFβ-treated NCI-H358 mesenchymal cells showed significantly enhanced sensitivity to BPTES relative to the untreated cells (Fig. 3D). The TGFβ-treated line had a 1.5-2 fold reduced growth rate compared to the parental line, though from the larger panel of lines screened, there was no correlation between growth rate and sensitivity to BPTES. This data, combined with the expression analyses of the cell line panel, strongly argues that mesenchymal lung cancer cells are predisposed to dependence on GLS1.

**Figure 4:**
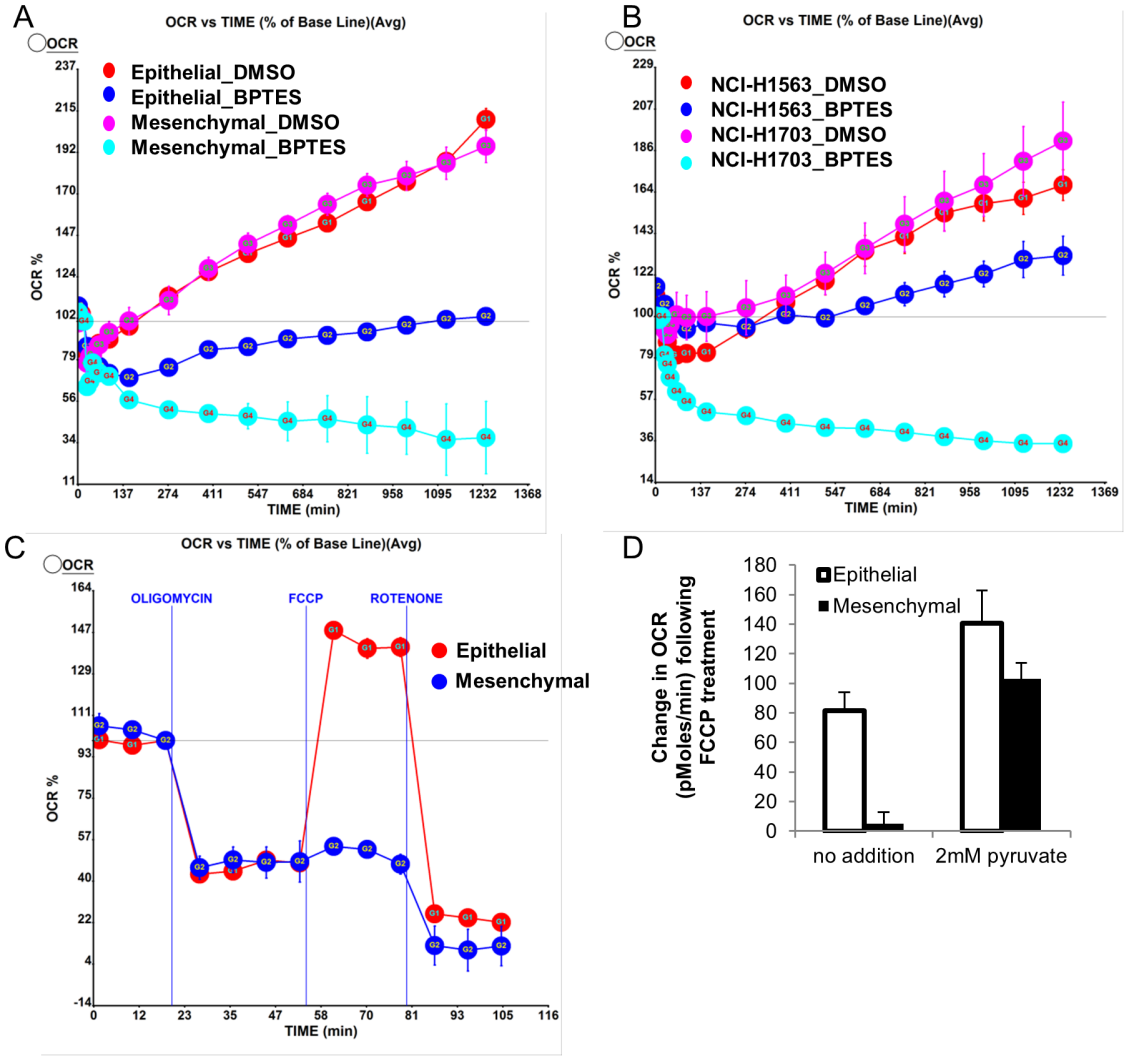
Oxygen consumption rates differentially altered upon drug perturbation in sensitive compared to insensitive cells. (A) NCI-H358 epithelial and mesenchymal lines or (B) NCI-H1703 (BPTES sensitive) and NCI-H1563 (BPTES insensitive) cells were treated with DMSO or 8 µM BPTES and oxygen consumption rate (OCR) was monitored over a 20 hr treatment. Data normalized to OCR at time zero (100%) and presented as mean values +/−SEM. *P* = 0.024 comparing changes in oxygen consumption with BPTES in the epithelial vs mesenchymal line; *P* = 0.00017 comparing NCI-H1703 and NCI-H1563 cells. (C) OCR following treatment with the indicated drugs to perturb mitochondrial respiration. Data normalized to OCR measurement prior to oligomycin treatment. (D) Addition of pyruvate to media restores the impaired FCCP response in the mesenchymal line. Average values +/−SEM indicated. Results representative of 2–3 independent experiments.

Since glutaminase serves as a key regulator of glutamine carbon entry into the TCA, we asked whether inhibition of GLS1 differentially affects oxidative phosphorylation (OXPHOS) in the epithelial compared to mesenchymal (NCI-H358_M) line. Mitochondrial oxygen consumption was measured in real time following BPTES treatment. At baseline, O_2_ consumption rate (OCR) was not significantly different between the two lines (S5A Figure in S1 File). Following 2 hr treatment with BPTES, OCR was comparably reduced in the epithelial and mesenchymal lines compared to the DMSO treated cells (Fig. 4A; S5B Figure in S1 File). Notably, with increasing time post-BPTES treatment, the OCR in the parental line slowly recovered, while OCR continued to decrease with time in the NCI-H358_M cells (Fig. 4A, S5B Figure in S1 File). The decrease in OCR was not reflective of decreased cell number at this early (20 hr) time point (S5C Figure in S1 File). To confirm that this effect of GLS inhibition was not unique to the NCI-H358 cells we performed the same experiment in an additional pair of sensitive (NCI-H1703) and insensitive (NCI-H1563) cells. Inhibition of O_2_ consumption was highly selective for the GLS1 dependent NCI-H1703 line (Fig. 4B) suggesting that impairment of oxidative phosphorylation is one component of the anti-proliferative effects induced by GLS inhibition.

The differential impact of BPTES on mitochondrial respiration in the mesenchymal versus epithelial line led us to question whether these lines might more broadly differ in their capacity to adaptively respond to metabolic/mitochondrial stress. To explore such a possibility, we performed sequential treatment of cells with oligomycin (ATP synthase inhibitor), FCCP (uncouples respiration from ATP synthesis, allows assessment of maximal respiration), and rotenone (inhibits entry of electrons at Complex I) [23]. Strikingly, the NCI-H358_M cells were defective in increasing O_2_ consumption in response to FCCP, indicative of a lower respiratory capacity of these cells (Fig. 4C; S5D Figure in S1 File). Recently, it was demonstrated that pyruvate is required for stimulation of maximal OCR in breast cancer cells [24]. Interestingly, additional supplementation of the RPMI media (containing 11 mM glucose and 2 mM glutamine) with 2 mM pyruvate in the presence of mitochondrial inhibitors restored the ability of the NCI-H358_M cells to respond to FCCP with an increased OCR (Fig. 4D), indicating that these cells may be unable to effectively utilize endogenous supplies of pyruvate/other alternative carbon sources to adequately fuel their reserve respiratory capacity. Taken together, these results suggest that the transition to a mesenchymal state may result in an impaired ability to adaptively respond to GLS inhibition and potentially to other mitochondrial stresses.

**Figure 5:**
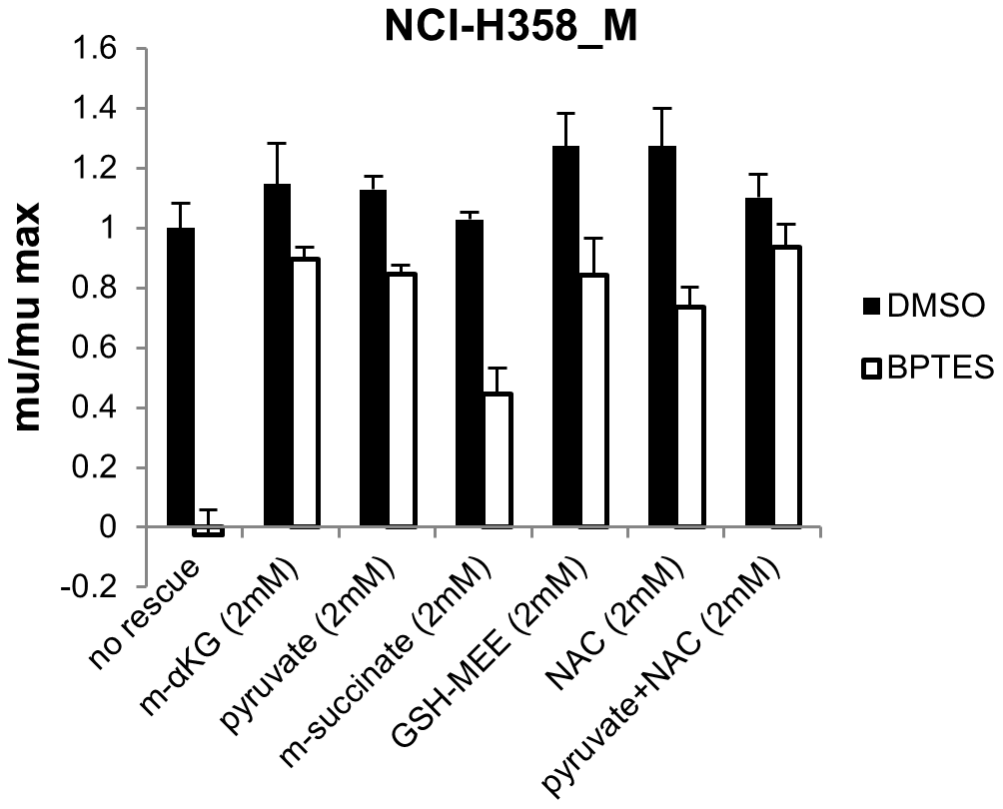
Rescue of anti-proliferative effects of BPTES by alternative carbon sources. NCI-H358_M cells were treated with DMSO or 8 µM BPTES in triplicate in the presence or absence of dimethyl 2-oxoglutarate (m-α-KG), sodium pyruvate, dimethyl succinate, glutathione reduced ethyl ester (GSH-MEE), or N-acetylcysteine (NAC) for 96 hrs and cell growth was measured by CTG. Results representative of 2 independent experiments. Values depict mean growth rate +/−SD relative to cells treated with DMSO alone. *P* values comparing DMSO vs BPTES treatment with the various rescue conditions: *P* = 0.0001 (no rescue), *P* = 0.04 (m-α-KG), *P* = 0.001 (2 mM pyruvate), *P* = 0.0003 (m-succinate), *P* = 0.01 (GSH-MEE), *P* = 0.003 (NAC), *P* = 0.06 (pyruvate + NAC).

We next tested whether alternative carbon sources could also rescue the impaired growth of the NCI-H358_M cells following BPTES treatment. Addition of either sodium pyruvate or a cell permeable analog of α-KG, dimethyl-2-oxoglutarate (m-αKG), to the growth medium resulted in a rescue of proliferation to ∼85–90% that observed in vehicle treated cells, compared to ∼45% for dimethyl succinate (Fig. 5). Unlike αKG and pyruvate, succinate to our knowledge has not been reported to have anti-oxidant properties. Since glutamate derived from glutamine can feed into the glutathione (GSH) biosynthetic pathway and contribute to maintaining redox balance [25] (as well as fueling the TCA via conversion to α-KG) we also examined the effect of a cell permeable derivate of GSH, glutathione reduced ethyl ester (GSH-MEE), and the antioxidant N-acetylcysteine (NAC) on BPTES induced growth inhibition. Both GSH-MEE and NAC alone restored growth to ∼75–85% that of vehicle treated cells while the combination of pyruvate and NAC restored proliferation to close to 95% that of control cells (Fig. 5). These findings suggest that impairments in both redox stress and TCA cycle anaplerosis contribute to the anti-proliferative effects of GLS inhibition in these cells.

**Figure 6:**
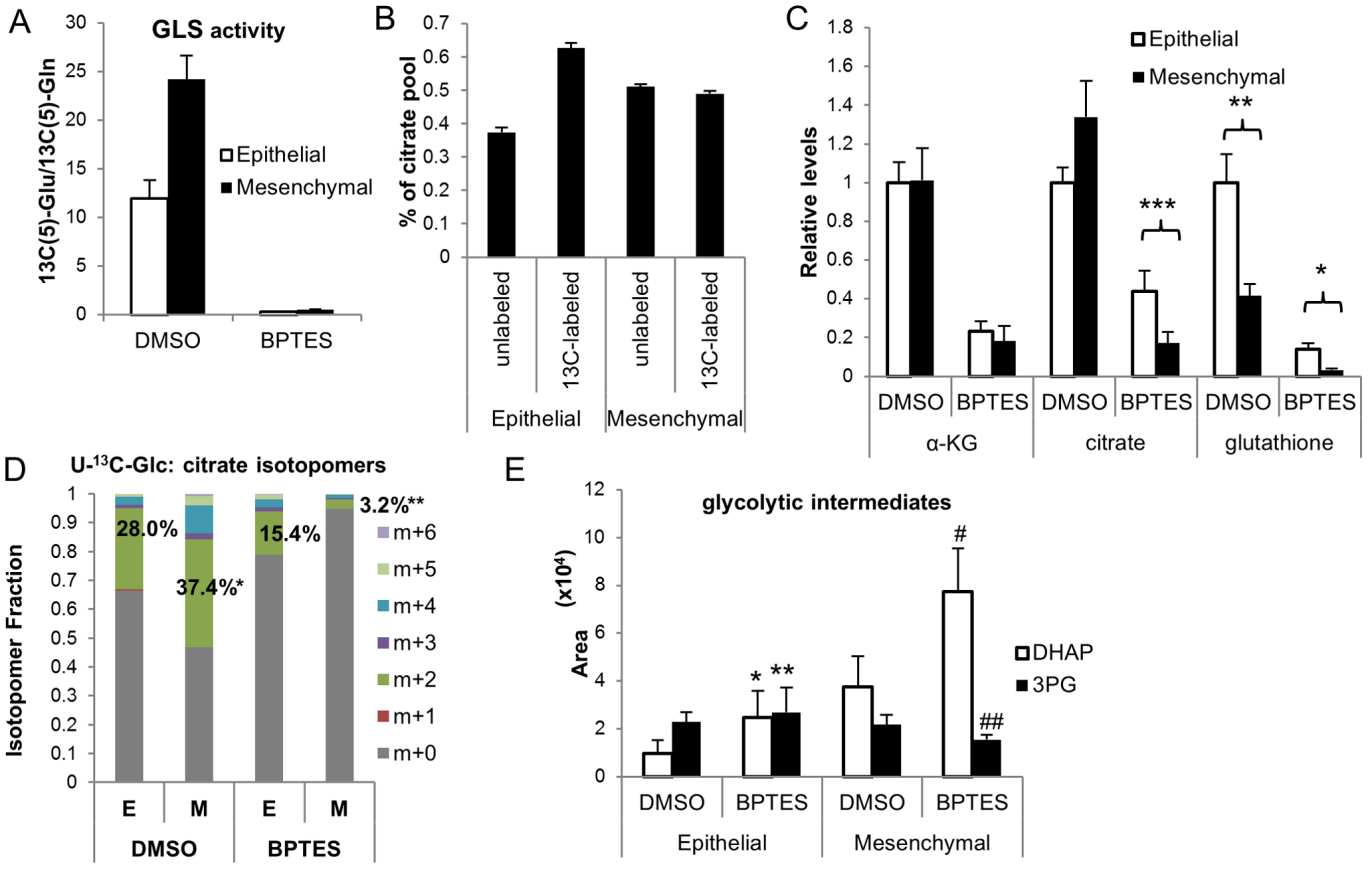
Metabolic profiling of epithelial and mesenchymal cells and effects of GLS inhibition. NCI-H358 epithelial (E) and mesenchymal (M) lines were treated with DMSO or 8 µM BPTES for 20 hrs. Cells were either unlabeled or U-^13^C-Glc or U-^13^C-Gln labels were included for the last 4 hrs of drug treatment and levels of central carbon metabolites were measured by LCMS. (A) Inhibition of GLS activity by BPTES. (B) Percentage of citrate pool that is either unlabeled or containing carbon derived from U-^13^C-Gln (DMSO treated cells). (C) TCA metabolite and glutathione pool sizes +/− BPTES. Data was collected in triplicate or quadruplicate from 3 independent experiments (for α-KG and citrate) and depicted as mean levels +/−SD relative to NCI-H358 DMSO treated cells; values were normalized for cell number; *P* =  *1×10^−5^; **4×10^−6^; ***9×10^−8^ (D) Citrate isotopomer percentages from ^13^C(6)-Glc labeled cells treated +/− BPTES. *P = 0.001, **P = 0.002 comparing % citrate m+2 in DMSO or BPTES treated E vs M cells, respectively. (E) Alterations in relative ratios of DHAP and 3PG levels in EvsM cells +/−BPTES. *P* values for comparison of metabolite levels +/−BPTES: *0.1, **0.57, ^#^0.04, ^##^0.07. Results representative of 2 independent experiments.

In order to further elucidate the mechanism underlying the differential sensitivity of the epithelial and mesenchymal NCI-H358 cells to GLS inhibition, we performed a metabolic analysis of these cells, examining patterns of glutamine and glucose (Glc) utilization (Glc labeling schematic depicted in S6 Figure (in S1 File) and the effects of GLS inhibition. We first confirmed that BPTES was comparably inhibiting GLS in both of the lines by monitoring ^13^C(5)-Gln and ^13^C(5)-Glu levels by LCMS in cells fed with uniformly labeled ^13^C-Gln (U-^13^C-Gln). Using ^13^C(5)-Glu/^13^C(5)-Gln levels as a readout for GLS activity, we demonstrated effective (97%) inhibition of GLS activity in both epithelial and mesenchymal lines (Fig. 6A; S7 Figure in S1 File). This data indicates that the vast majority of conversion of Gln to Glu in these cell lines is catalyzed by GLS1 and not GLS2 or other Gln utilizing enzymes. One potential hypothesis to explain the differential sensitivity of the two lines was that the mesenchymal cells might preferentially utilize glutamine to fuel the TCA. Surprisingly, a 4 hr feed of cells with U-^13^C-Gln demonstrated substantial contribution of glutamine to citrate pools in both the epithelial (63%) and mesenchymal (49%) line (Fig. 6B). Consistent with the prominent contribution of glutamine to citrate pools, a 20 hr BPTES treatment reduced the total levels of TCA metabolites, including α-KG and citrate (Fig.6C; S7 Figure in S1 File). Little or no effect of drug on cell numbers was observed within this time of BPTES treatment in these (S5C Figure in S1 File) and other cell lines tested (S1 Figure in S1 File). In spite of the fact that glutamine contributed more prominently to citrate pools in the epithelial compared to mesenchymal line (Fig. 6B), citrate levels dropped more dramatically and to lower (61%) total levels in the BPTES treated NCI-H358_M cells. Taken together with the increased drop in O_2_ consumption by BPTES in the mesenchymal compared to epithelial line, these data suggest a potential impairment in the ability of the mesenchymal line to adapt to a block of Gln flow into the TCA, perhaps due to a differential ability to use alternative carbon sources in this setting.

Interestingly, monitoring of glucose flow into the TCA using a U-^13^C-Glc feed (S7 Figure in S1 File), revealed glucose contribution to 28% and 37% of the total citrate pool in epithelial and mesenchymal cells, respectively; upon BPTES treatment, this relative contribution was reduced to 15% in the epithelial and 3% in the mesenchymal line (Fig. 6D; S7 Figure in S1 File), indicative of a selective block in glucose flow into the TCA with BPTES treatment in the mesenchymal line. Consistent with this notion, examination of glycolytic intermediate pool sizes revealed increased DHAP and reduced 3-PG levels (Fig. 6E; S7 Figure in S1 File) with BPTES treatment in the mesenchymal line, indicating a block in flow at this step in glycolysis.

In addition to differences in metabolites involved in bioenergetic/biosynthetic pathways, the NCI-H358_M cells had lower reduced glutathione (GSH) levels in both the basal and BPTES treated setting compared to the epithelial variant (Fig. 6C; S7 Figure in S1 File). In 6 additional NSCLC lines examined, there was a similar trend between degree of drop in GSH pools with BPTES sensitivity (S8A Figure in S1 File), leading us to investigate the effects of BPTES treatment on ROS levels.

### Modulation of oxidative stress by GLS inhibition in mesenchymal NSCLC cells correlates with sensitivity

**Figure 7:**
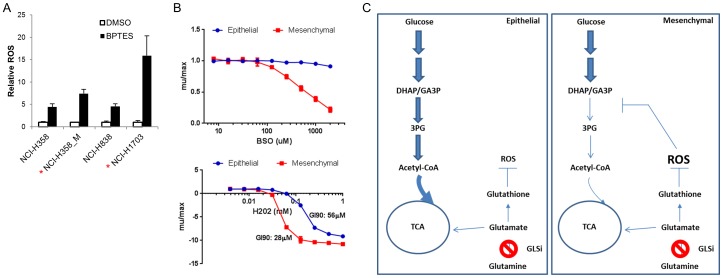
Reduced capacity to cope with oxidative stress in mesenchymal/BPTES sensitive cells. (A) Cells were treated with BPTES for 20 hrs and ROS levels measured with CM-H2DCFDA dye. Results representative of 3 independent experiments; data plotted as ROS levels relative to DMSO treatment of each cell line (mean +/−SD); *P* = 0.01 comparing induction of ROS by BPTES in the 2 pairs of sensitive/insensitive lines. (B) NCI-H358 epithelial and mesenchymal lines were treated with BSO or H_2_0_2_ in a 72 hr CTG assay. Results representative of 2-3 independent experiments and plotted as mean +/-SD. (C) Model for sensitivity of mesenchymal NSCLC cells to GLS inhibition. Inhibition of Gln→Glu conversion by BPTES impairs flow of carbon from Gln into the TCA in both lines. In the mesenchymal line, BPTES treatment results in a selective block in glucose flow into the TCA due to impaired flow from DHAP/GA3P to 3-PG in upper glycolysis. A model is proposed wherein increased ROS levels following BPTES treatment in the mesenchymal line underlie the altered flow through glycolysis.

To test whether sensitivity to GLS inhibition correlated with increased oxidative stress upon BPTES treatment, we measured levels of reactive oxygen species (ROS) using CM-H2DCFDA in the NCI-H358 epithelial and mesenchymal pair as well as another pair of BPTES sensitive (NCI-H1703)/insensitive (NCI-H838) lines. The NCI-H838 line was chosen for examination since, similar to the NCI-H358 line, it exhibits poor sensitivity to BPTES despite having a substantial contribution of Gln to TCA carbon pools (unpublished data). While GLS inhibition resulted in increased ROS levels in all four lines tested, the sensitive lines exhibited a greater magnitude of increase in ROS by BPTES (7.4 vs 4.4-fold in NCI-H358_M compared to parental; 15.9-fold vs 4.6-fold in NCI-H1703 compared to NCI-H838 cells; Fig. 7A). Interestingly, it has been shown that oxidative stress can impair carbohydrate flux through glycolysis via inactivation of enzymes, including GAPDH [26]. Taken together, these data suggest a model in which increased ROS generation following GLS inhibition in the mesenchymal cells sensitizes to GLS inhibition in part via inhibition of glucose flow into TCA, resulting in reduced citrate levels and OXPHOS (Fig. 7C).

We next asked whether other means of reducing glutathione/increasing oxidative stress differentially affects the epithelial and mesenchymal NCI-H358 cells. Interestingly, the mesenchymal line was significantly more sensitive than the epithelial line to perturbation of GSH synthesis with the glutamylcysteine transferase inhibitor buthionine sulfoximine (BSO). The maximum dose of BSO used (2 mM) resulted in a 79% reduction in growth rate in the NCI-H358_M cells compared to a 9% reduction in growth rate in the parental line. Examination of two additional pairs of BPTES sensitive (A427, NCI-H1703) and insensitive (NCI-H838, NCI-H1563) lines revealed a similar pattern of increased sensitivity to BSO in the BPTES sensitive lines (S8B Figure in S1 File). Similarly, lower concentrations of hydrogen peroxide were required to blunt growth/kill NCI-H358_M compared to parental cells (GI90 = 28 vs 56 µM; Fig. 7B). To confirm that the increased sensitivity of the mesenchymal cells to GLS inhibition and oxidative stress inducing agents was not simply indicative of a heightened sensitivity to all drug treatments, the two lines were examined for sensitivity to the EGFRi, gefitinib as well as the chemotherapeutic agent, docetaxel. In contrast to the GLS inhibitor and oxidative stress inducing agents, gefitinib and docetaxel were more effective at inhibiting growth in the epithelial compared to mesenchymal NCI-H358 cells (S9 Figure in S1 File).

## Discussion

The recent explosion in cancer metabolism research has focused not only on the area of glucose and the Warburg effect, but also on the complementary roles of myriad other nutrients that contribute to cancer cell growth. Glutaminase has emerged as one of several enzymes utilized by tumors to enable Gln use as a key nutrient for some tumor cells. As a result, targeting glutaminase as a cancer therapeutic has garnered significant attention. To our knowledge, *GLS1* is not consistently mutated or amplified in any reported cancers. Recent studies have highlighted VHL-deficient RCC [27] and IDH1 mutant glioblastoma [12] as particular genetic settings in which tumors may depend on GLS. In an analysis of Gln dependency in NSCLC, it was found that cell lines varied widely in their reliance on Gln, although there were no clear genetic markers identified that correlated with this dependence, including GLS1 expression levels [7]. As Gln is utilized for nucleotide synthesis and glycosylation pathways [28], in addition to fueling the TCA cycle via GLS activity, it is likely that multiple factors play a role in determining Gln dependence. In this study, we screen a panel of lung cancer lines with a published tool compound, BPTES, to specifically inhibit Gln utilization via GLS1. Although BPTES has been used extensively in the literature, prior to this study, it had not been validated for the specificity of its cellular effects. We herein validate the specificity of the cellular and metabolic effects of BPTES and demonstrate, for the first time, that a mesenchymal phenotype predisposes NSCLC cells to dependence on GLS1.

In addition to being marked by low E-cadherin/high vimentin expression, BPTES sensitive NSCLC lines on average had elevated GAC and lower pyruvate carboxylase expression compared to insensitive lines. Notably, GAC expression was selectively increased with TGFβ treatment in the NCI-H358 NSCLC model. Together these findings represent an interesting extension of previous findings in which TGFβ was found to enhance the expression of GLS1 in the LLC-PK(1)-FBPase model system [29]. Further, in a comprehensive study by Thomson et al [30] examining the downstream consequences of EMT, GLS expression was found to be similarly elevated at the transcriptional level in NCI-H358 cells that were induced to undergo EMT via several different mechanisms (TGFβ treatment, or SNAIL/ZEB1 overexpression). Another recent study examining metabolic gene alterations in several pairs of non-invasive epithelial vs. invasive mesenchymal breast cancer lines reported elevated GLS1 gene transcription in the mesenchymal versus epithelial lines [31]. Taken together with our work, these data indicate that TGFβ/EMT may be a regulator of glutaminase and metabolic control in multiple diverse systems.

The possibility that the process of EMT results in altered metabolic dependencies is of particular interest given the association between EMT and acquired resistance to a growing list of targeted therapies in a number of cancer types [32]–[35]. Conversely, several recent studies have shown that alterations in metabolism can regulate EMT. Knockdown of ATP citrate lyase was shown to reverse EMT in lung cancer cells [36], while knockdown of citrate synthase was found to induce EMT [33]. Also interesting is the recent elucidation of a stem or mesenchymal cellular program that is seen in the context of IDH1/2 mutations downstream of epigenetic regulation by 2-hydroxyglutarate [37], [38]. Ongoing studies should clarify if it is the EMT program that drives sensitivity to GLS1 inhibitors in these settings. The implication that a GLS1 inhibitor might be used for the treatment of a subset of NSCLC with mesenchymal markers is an exciting prospect since this tumor type is generally described as being late stage, aggressively metastatic, and highly refractory to current therapeutic strategies [34].

Many questions remain to be answered, including whether a mesenchymal phenotype predisposes to GLS inhibition in the *in vivo* setting. A more thorough understanding of the molecular mechanisms that underlie GLS dependence and through which cells can potentially compensate for loss of GLS activity will guide in the investigation of relevant combination strategies. Our data suggests that a reduced capacity to cope with oxidative stress in association with EMT may in part dictate the response to GLS inhibition in the mesenchymal NSCLC models examined. Rescue experiments in additional sensitive lines will help clarify the general importance of oxidative stress induction to the growth inhibitory phenotype. As both the thioredoxin/peroxiredoxin and glutathione pathways play an important role in maintaining redox balance [39], it is notable that reduced peroxiredoxin 2/5 and thioredoxin levels were observed upon EMT in the OSI study described above [30]. In such a setting cells may be particularly vulnerable to perturbations in the glutathione pathway driven by GLS inhibition or BSO treatments. Since the generation of ROS can regulate TGF-β signaling and its effects on differentiation/EMT [40]–[42], the possibility remains that alterations in redox state could serve as an initial driver and link between mesenchymal phenotype and GLS1 dependence. Interestingly, in the context of DNA damage/oxidative stress, induction of GLS2 expression has been shown to occur in a p53-dependent manner, indicating that both GLS1 and GLS2 are positioned to protect cells from pathways that induce oxidative stress [43].

Studies are currently underway to achieve a better understanding of the molecular mechanisms driving altered dependence on GLS in the setting of EMT, as well as to achieve a more comprehensive analysis of alterations in metabolite flux that occur with this transition. In particular, it will be interesting to determine if the BPTES mediated reduction in citrate levels differentially impacts lipid biosynthesis in the mesenchymal compared to epithelial cells. Since pyruvate carboxylase levels negatively correlate with BPTES sensitivity, differences in the ability to adapt to GLS inhibition by oxidation of glucose could be a factor in the more dramatic drops in respiration and growth inhibition in sensitive versus insensitive lines. It is tempting to speculate that the substantial increase in oxidative stress that occurs following GLS inhibition in sensitive lines can modulate enzymes such as GAPDH, a known target of oxidative modification [44], [45], impairing glycolytic flux [26]. Finally, future studies will help establish whether or not GLS1 dependence is driven by EMT and oxidative stress in other tumor types where EMT has been shown to play an important role in tumorigenesis [46]. Recent studies have suggested that glutamine and GLS1 activity are required for optimal growth of breast cancer cell lines *in vitro* and *in vivo* [47], [48] and additional work will clarify whether mesenchymal markers predict sensitivities to GLS inhibition in this population.

## Materials and Methods

### Cell Culture

All cell lines described in these studies were obtained from the American Type Culture Collection (ATCC, Manassas, VA) or the Deutsche Sammlung von Mikroorganismen und Zellkulturen (DSMZ, Hamburgn, Germany). Cells were routinely cultured in RPMI1640 containing 25 mM Hepes and 2 mM L-Glutamine (Lonza, Walkersville, MD) supplemented with 10% Fetal Bovine Serum (Thermo Scientific), 100U/ml penicillin and 100ug/ml streptomycin (Life Technologies) in an atmosphere of 5% CO_2_ and 37°C. A427 cells expressing constitutively active GAC and mutant GAC [GAC-BR (F318A and F322A)] were maintained in standard growth media supplemented with 0.3ug/ml Geneticin (Life Technologies). NCI-H358 cells were induced to undergo EMT by treatment with 25ng/ml TGF-β3 (Peprotech, Rocky Hill, NJ). All mechanistic experiments were carried out with cells that had been treated with TGF-β3 for 6-10 weeks.

### Compounds

BPTES and AGX-4769 were synthesized by SAI Life Sciences (Hyderabad, India). Gefitinib and docetaxel were purchased from Selleckchem (Houston, Texas) and Sigma (St Louis, Missouri), respectively.

### Western Blot Analyses

An antibody recognizing GLS1 and GAC splice variants was generated using the human GLS1/GAC peptide sequence GPKDGPGETDAFGNSEGK (aa 104-121), followed by affinity purification (YenZym Antibodies LLC, San Francisco, CA). The peptide used as an immunogen represents a sequence not conserved in human GLS2. E-cadherin, vimentin, β-catenin, and histone H3 antibodies were purchased from Cell Signaling Technology (#s 3195, 5741, 9582, and 4499 respectively), β-actin antibody was purchased from Sigma-Aldrich (A1978), and pyruvate carboxylase antibody was purchased from Proteintech Group Inc (16588-1-AP). Quantitative western blots were obtained with the LI-COR Bisosciences (Lincoln, NE) Odyssey infrared imaging system.

### Viability Assays

Cells (2-5K) were plated into black, clear-bottom, tissue cultured treated, 96-well plates in standard growth medium. To measure sensitivity to GLS inhibition, media was removed 18–24 hrs post plating and replaced with RPMI1640 containing 5 mM glucose and 0.5 or 2 mM L-glutamine, and BPTES or AGX-4769 at a starting concentration of 10 µM. Serial dilutions were performed in media containing 0.1% DMSO. Three replicates of each drug concentration were performed. Cell viability was measured using CellTiter-Glo Luminescent Cell Viability Assay (Promega, Madison, WI). CellTiter-Glo assays were performed at the time of drug addition (t0) and 72 hrs post drug addition (t72). Growth rates (mu) were calculated using the formula: [LN(T72/T0)]/time(hrs); µ/µmax calculations were used to compare growth rates of drug treated to DMSO treated cells (µ_BPTES_/µ_DMSO_). The assay was repeated a minimum of two times for each cell line. The effects of L-Butathionine-sulfoximine and hydrogen peroxide (Sigma) treatment on cell growth were similarly assessed. For Syto60 assays, cells were washed with PBS, fixed with 4% paraformaldehyde, and stained with 0.625 µM Syto60 (Life Technologies) for 30 min at room temperature. Cells were washed with PBS and fluorescence intensity measured on a SpectraMax (Molecular Devices) using SoftmaxPro software. For BPTES rescue experiments, agents were added concomitant with drug treatment. Dimethyl 2-oxoglutarate, dimethyl succinate, glutathione reduced ethyl ester, and N-acetylcysteine were all purchased from Sigma; sodium pyruvate was purchased from Lonza.

### Generation of A427 Engineered Lines

A427 cells were infected with pLVX retroviral vectors containing a neomycin resistance cassette and either full-length wild-type human GAC cDNA or GAC-F318A, F322A mutations (GAC-BR)[16]. GAC cDNA constructs were untagged to avoid interference with normal function and localization of the enzyme.

### siRNA Transfections

A427 cells were plated in 6 cm dishes to achieve ∼30% confluence at the time of transfection. Cells were transfected with a non-targeting (NT) siRNA pool (5′-UGGUUUACAUGUCGACUAA-3′, 5′-UGGUUUACAUGUUGUGUGA-3′, 5′-UGGUUUACAUGUUUUCUGA-3′, 5′-UGGUUUACAUGUUUUCCUA-3′), or a pool of 4 CTNNB1 targeting siRNAs (5′-GAUCCUAGCUAUCGUUCUU-3′, 5′-UAAUGAGGACCUAUACUUA-3′, 5′-UAAUGAGGACCUAUACUUA-3′, 5′-GGUACGAGCUGCUAUGUUC-3′; ON-Target plus SMARTpool (Dharmacon), using Lipofectamine RNAiMAX (Life Technologies). Reagents were diluted in OPTI-MEM and transfection complexes were added to cells in antibiotic-free media (10 nM final siRNA concentration). 24 hours post-transfection, cells were harvested and plated at 2K/well in 96-well plates for a 72 hr proliferation assay. Protein extracts were harvested 72 hours post-transfection for assessment of KD efficiency.

### Bioinformatics Analysis

The Broad cell line expression profiling dataset (caArray database, accession golub_00327, https://array.nci.nih.gov/caarray/project/details.action?project.id=327) was used to identify RNA species that are associated with BPTES sensitivity or resistance. For each probe set, the Pearson correlation (cor) coefficient was calculated between log2(MAS 5.0) signal and mu/mumax values (positive Pearson cor.values indicate an anti-correlation between expression and BPTES sensitivity). The top 200 correlated or anti-correlated probes were submitted to GeneGo pathway analysis (http://portal.genego.com/cgi/data_manager.cgi) to identify significantly enriched pathways. To identify individual EMT-related genes that significantly correlated with BPTES sensitivity, the list of correlated probes was filtered for genes that are altered during EMT, extracted from the Broad Molecular Signature Database (MsigDB_v4).

### Metabolomics Analyses

^13^C_5_-L-glutamine and ^13^C_6_-D-glucose were purchased from Sigma. For analysis of the on-target effects of BPTES on metabolite changes, A427 cells with no exogenous GLS1 expression, over-expressing GAC, or GAC-F322S/F318Y were plated into a 96-well tissue culture treated plate to a confluence of 50–60% in RPMI1640 containing 25 mM HEPES, 5 mM glucose, 0.5 mM L-glutamine, penicillin/streptomycin, and 5% dialyzed FBS, and incubated overnight at 37°C, 5% CO_2_. The cells analyzed grew equally well in 2 vs 0.5mM glutamine. 18–24hrs post plating, media was removed and the cells were pre-treated with media containing BPTES or AGI-4769 in 0.1% DMSO and incubated at 37°C, 5% CO_2_ for 1 hr. Three replicates of each compound concentration were performed. After 1 hr pre-treatment, media was removed and cells were incubated for another 2 hrs with media (containing BPTES or AGI-4769 at the same concentrations as pre-treatment) in which the unlabeled Gln was replaced with 0.5 mM ^13^C_5_- L-glutamine. Cells were washed once with 70 mM ammonium carbonate buffer pH 7.5 and metabolites were extracted with ice cold 80% methanol with L-Glutamic acid-d3 (1 µg/mL) as internal standard for mass spectrometric analysis. Cell debris was removed by centrifugation and samples were dried under a stream of nitrogen air.

Cell extracts were analyzed by liquid chromatography mass spectrometry (LC-MS) in negative mode using selective reaction monitoring (SRM) for each metabolite of interest. Prior to injection, dried extracts were reconstituted in LCMS grade water. LC separation was achieved by using a Waters T3 column: 1.7 µm, 50 mm×2.1 mm i.d. and an Acquity UPLC system with a gradient mobile phase of 5% methanol in water containing 5 mM acetic acid and 10 mM tributylamine (Solvent A) and methanol (Solvent B) for 3.2 min at a flow rate of 0.4 mL/min. The gradient started with 5% B (0–0.5 min); 5% B to 70% B (0.5–1.0 min); 70% B held for 0.2 min (1.0–1.2 min); 70% B to 95% B (1.2–1.3 min); 95% B held for 0.7 min (1.3–2.0 min); 95% B to 5% B (2.0–2.2 min); 5% B held for 1 minutes (2.2–3.2 min) to re-equilibrate the column.

For analysis of metabolic differences in the NCI-H358 epithelial and mesenchymal lines, cells were plated as above and treated with 8 µM BPTES/0.1%DMSO for 20 hrs. For assessment of pool size changes, extracts were prepared and analyzed as above. For glucose and glutamine tracking, cells were labeled with 0.5 mM ^13^C_5_-L-glutamine or 5 mM ^13^C_6_-D-glucose for the last 4 hrs of drug treatment (16 hrs post-initial drug treatment). Cell extracts were prepared from live cultures on Hamilton StarPlus system running an automated metabolite extraction protocol using hot 70% aq. Ethanol (70 C). Supernatant of extracted samples were then dried down on GeneVac EZ-2 evaporator, followed by re-suspension for analysis. Cell extracts obtained as described above were analyzed for relative abundance of ^13^C metabolites by liquid chromatography-mass spectrometry (LC-MS) in negative (as described above) and positive mode.

For acquisition of metabolites in positive mode, high resolution accurate mass (HRAM) LC-MS analysis was employed. The U-HPLC system consisted of a Thermo Fisher Scientific (San Jose, USA) U-HPLC pumping system, coupled to an autosampler and degasser. Chromatographic separation of the intracellular metabolites was achieved by usage of a reversed phase Atlantis T3 (3 µm, 2.1 mm ID×150 mm) column (Waters, Eschborn, Germany) and by implementation of a gradient elution program. The elution gradient was carried out with a binary solvent system consisting of 0.1% acetic acid and 0.025% heptafluorobutyric acid in water (Solvent A) and in acetonitrile (Solvent B) at a constant flow rate of 400 µL min^−1^. The linear gradient employed was as follows: 0–4 min increase from 0 to 30% B, 4–6 min from 30 to 35% B, 6–6.1 min from 35 to 100% B and hold at 100% B for 5 min, followed by 5 min of re-equilibration. The column oven temperature was maintained at 25°C and sample volumes of 10 µL were injected. HRAM data was acquired using a QExactive Orbitrap mass spectrometer (Thermo Fisher Scientific), which was equipped with a heated electrospray ionization source (HESI-II), operated in positive mode. Ionization source working parameters were optimized; the heater temperature was set to 300°C, ion spray voltage was set to 3500 V. An *m*/*z* scan range from 70 to 500 was chosen and the resolution was set at 70,000. The automatic gain control (AGC) target was set at 1e^6^ and the maximum injection time was 250 ms. Instrument control and acquisition was carried out by Xcalibur 2.2 software (Thermo Fisher Scientific) and Tracefinder 2.1 software, respectively (Thermo Scientific). Data acquired were processed using Metabolomic Analysis and Visualization Engine for LC-MS data (MAVEN) [49].

### Oxygen Consumption Measurements

For measurement of mitochondrial respiration, cells were plated at 40K/well in Seahorse XF24 plates in standard culture media. The following day, media was changed to unbuffered RPMI (Sigma R8755) containing 10% dialyzed FBS and respiration monitored with the Seahorse Bioscience XF24 instrument. Three baseline measurements were taken prior to injection with drug. For the mitochondria stress test, drugs were injected to give final concentrations of 5 µM (oligomycin, FCCP) or 10 µM (rotenone). For each well, oxygen consumption rate (OCR) values were normalized so that pre-treatment levels  = 100%. Total oxygen consumption over the course of the assay was estimated by calculating the area under the normalized OCR curve. Oxygen consumption readouts for the two BPTES-treated cell lines were compared by t-test.

### Reactive Oxygen Species Measurements

Cells were plated at 8-10K/well in black-walled 96-well plates in standard growth media. 18-24 hrs post-plating, cells were treated with 8 µM BPTES/0.1%DMSO in phenol red-free RPMI/10% FBS for 20 hrs. CM-H2DCFDA (Life Technologies) was added to cells at a final concentration of 5 µM in phenol red-free RPMI (in the absence of serum). Following a 30 min incubation with dye, media was replaced with phenol red-free RPMI/10% FBS and cells were left to recover for an additional 30 min prior to washing with PBS and fluorescence measurement on a SpectraMax plate reader.

## Supporting Information

S1 File **Combined file of supporting figures, legends, and methods.**
(PDF)

S2 File **Combined file of supporting tables.**
(XLSX)
