# Supplementary material for: Mesenchymal Phenotype Predisposes Lung Cancer Cells to Impaired Proliferation and Redox Stress in Response to Glutaminase Inhibition
Source: PLoS One. 2014 Dec 12;9(12):e115144. doi: 10.1371/journal.pone.0115144 (PMC4264947; doi:10.1371/journal.pone.0115144)
Supplement: S1 File — Combined file of supporting figures, legends, and methods. (PDF) [file pone.0115144.s001.pdf]

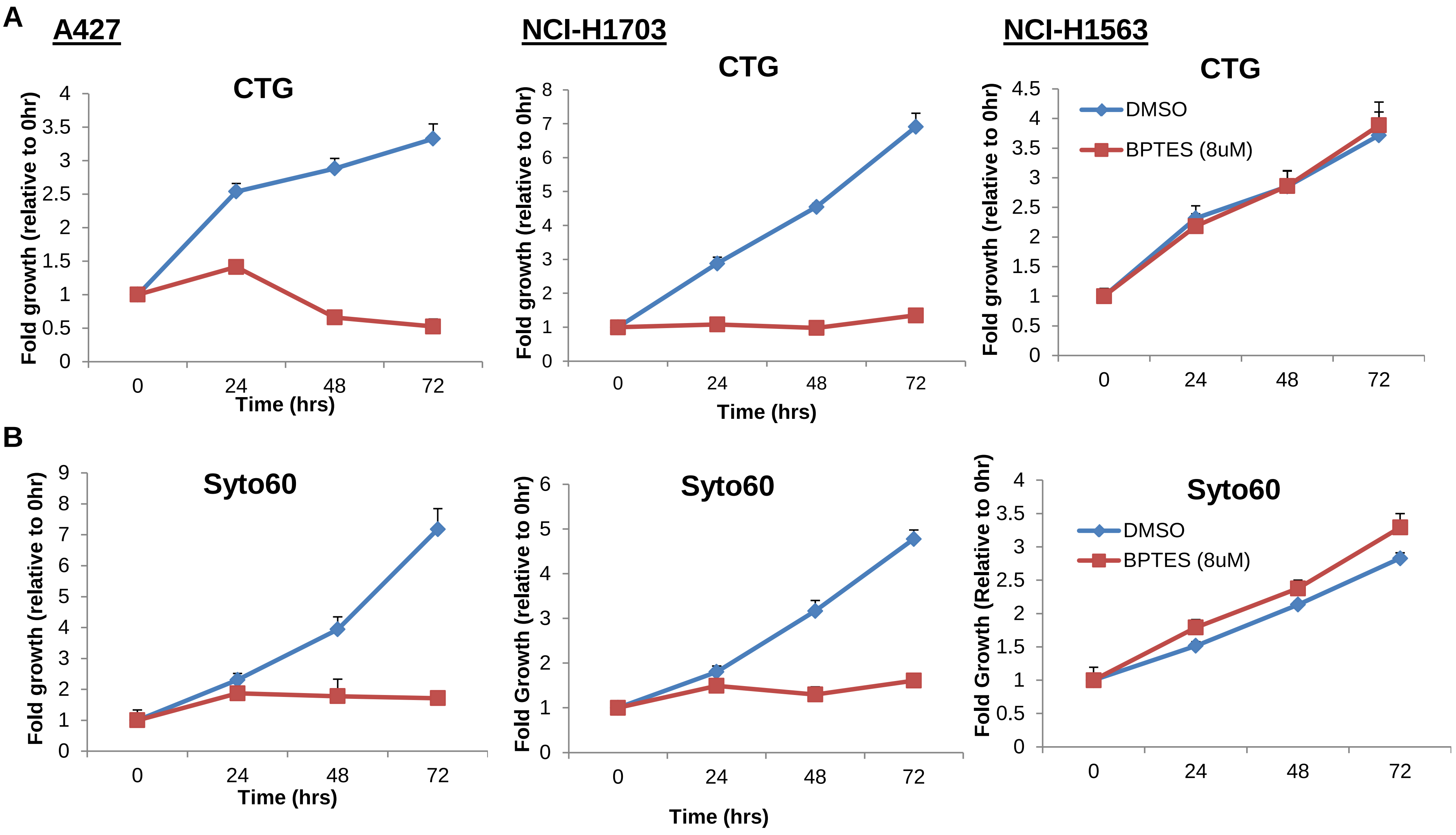
A
A427
CTG
Fold growth (relative to 0hr)
Time (hrs)
NCI-H1703
CTG
Fold growth (relative to 0hr)
Time (hrs)
NCI-H1563
CTG
DMSO
BPTES (8uM)
Fold growth (relative to 0hr)
B
Syto60
Fold growth (relative to 0hr)
Time (hrs)
Syto60
Fold Growth (relative to 0hr)
Time (hrs)
Syto60
DMSO
BPTES (8uM)
Fold Growth (Relative to 0hr)
0
24
48
72

Figure S1

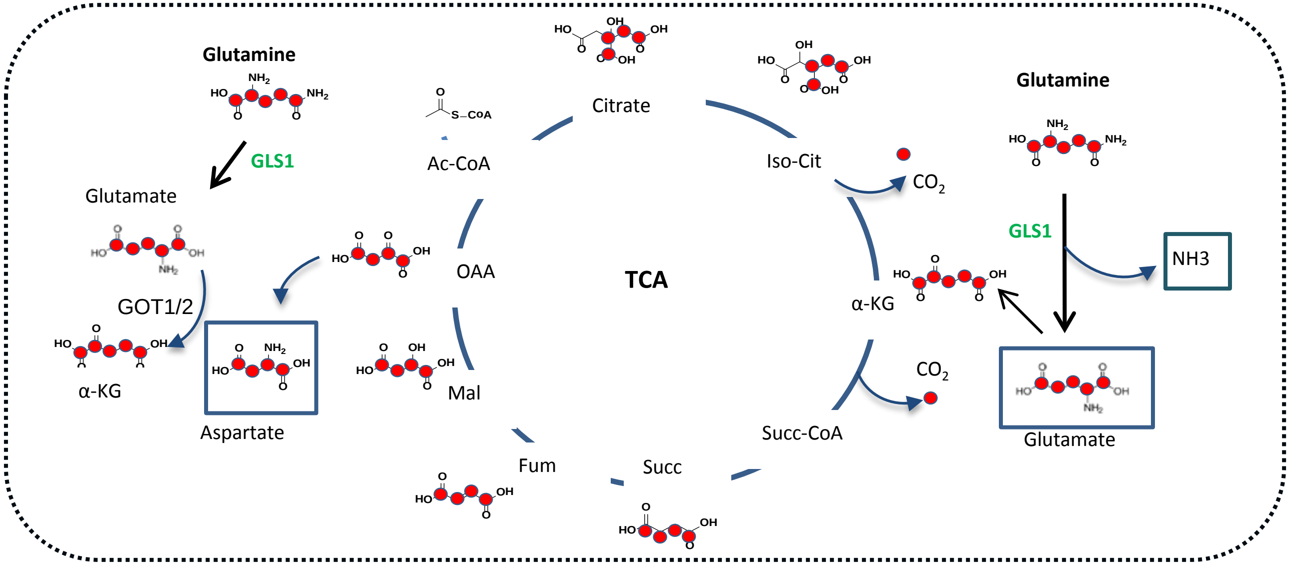

Glutamine
GLS1
Glutamate
GOT1/2
α-KG
Aspartate
Ac-CoA
Citrate
Iso-Cit
CO_2
OAA
TCA
α-KG
Mal
Fum
Succ
Succ-CoA
CO_2
Glutamine
GLS1
NH3
Glutamate

Figure S2

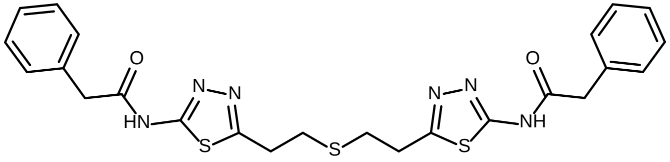

BPTES

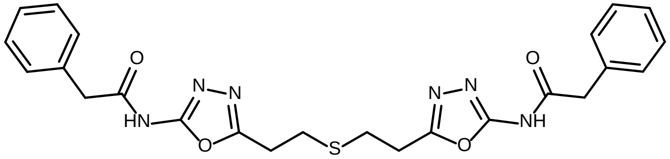

AGX-4769

Figure S3

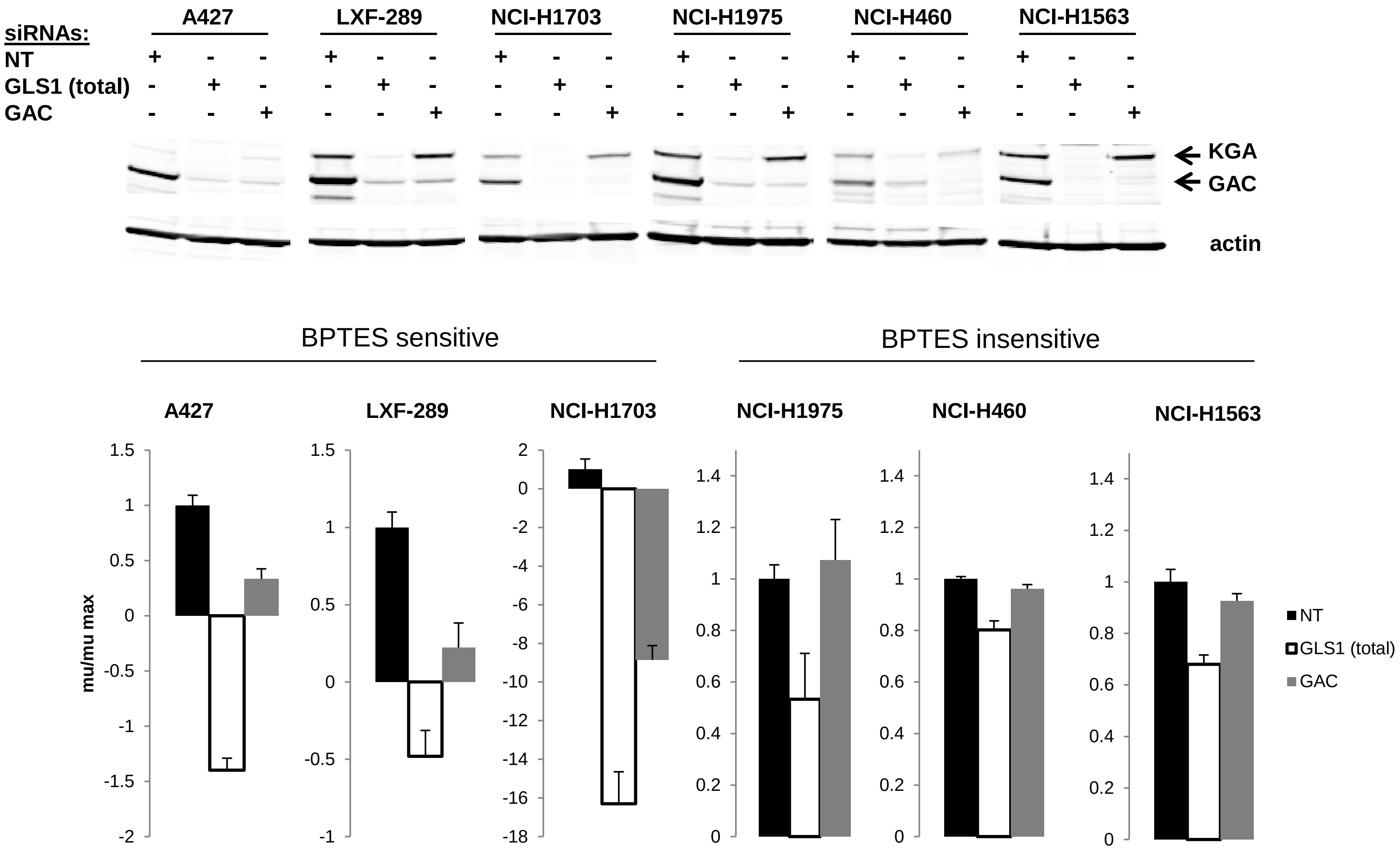

siRNAs:
NT
GLS1 (total)
GAC
A427
LXF-289
NCI-H1703
NCI-H1975
NCI-H460
NCI-H1563
KGA
GAC
actin
BPTES sensitive
BPTES insensitive
mu/mu max
NT
GLS1 (total)
GAC

Figure S4

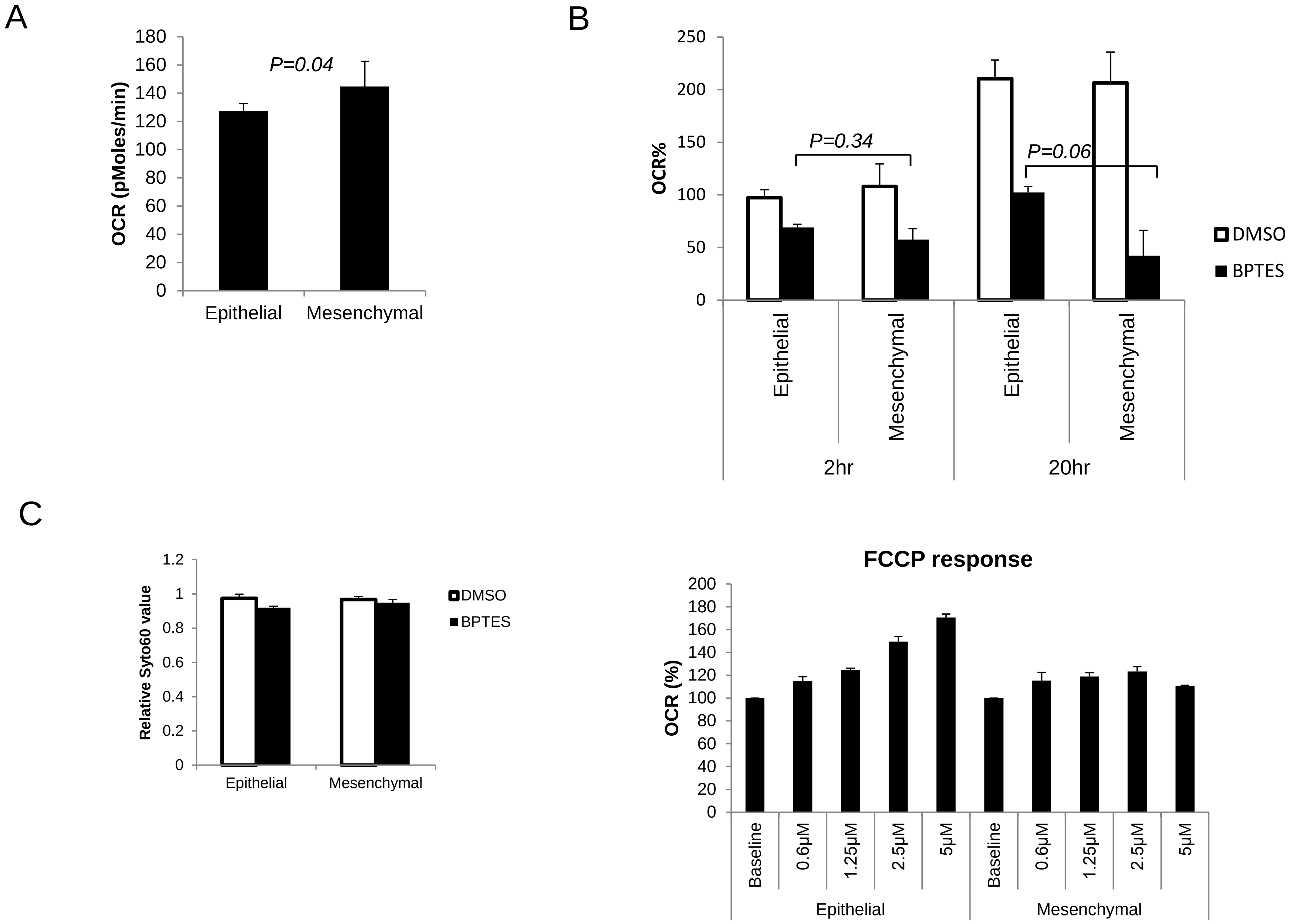
A
180
160
140
120
100
80
60
40
20
0
OCR (pMoles/min)
P=0.04
Epithelial
Mesenchymal
B
250
200
150
100
50
0
OCR%
P=0.34
P=0.06
DMSO
BPTES
Epithelial
Mesenchymal
Epithelial
Mesenchymal
2hr
20hr
C
1.2
1
0.8
0.6
0.4
0.2
0
Relative Syto60 value
DMSO
BPTES
Epithelial
Mesenchymal
FCCP response
200
180
160
140
120
100
80
60
40
20
0
OCR (%)
Baseline
0.6μM
1.25μM
2.5μM
5μM
Baseline
0.6μM
1.25μM
2.5μM
5μM
Epithelial
Mesenchymal

Figure S5

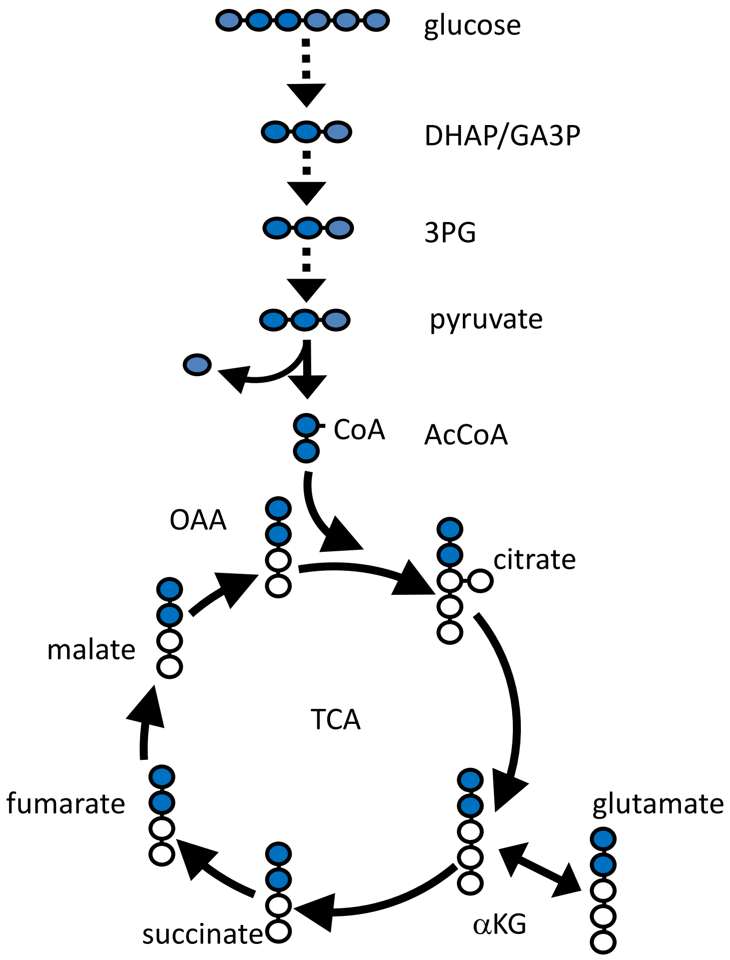
glucose
DHAP/GA3P
3PG
pyruvate
CoA
AcCoA
OAA
citrate
malate
TCA
fumarate
glutamate
succinate
αKG

Figure S6

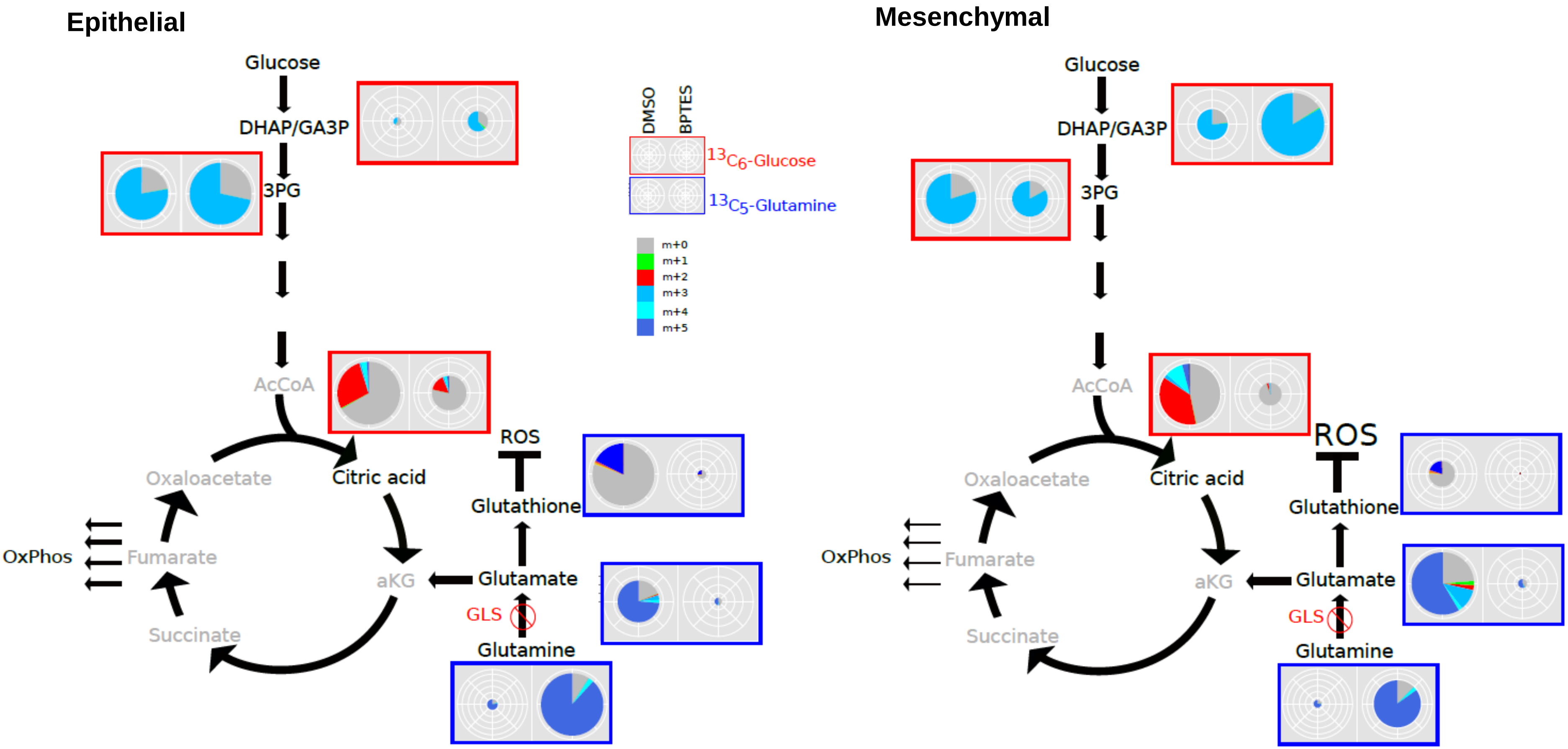

Epithelial
Mesenchymal
Glucose
DHAP/GA3P
3PG
AcCoA
Citric acid
Oxaloacetate
Fumarate
Succinate
aKG
OxPhos
ROS
Glutathione
Glutamate
GLS
Glutamine
DMSO
BPTES
13C6-Glucose
13C5-Glutamine
m+0
m+1
m+2
m+3
m+4
m+5

Figure S7

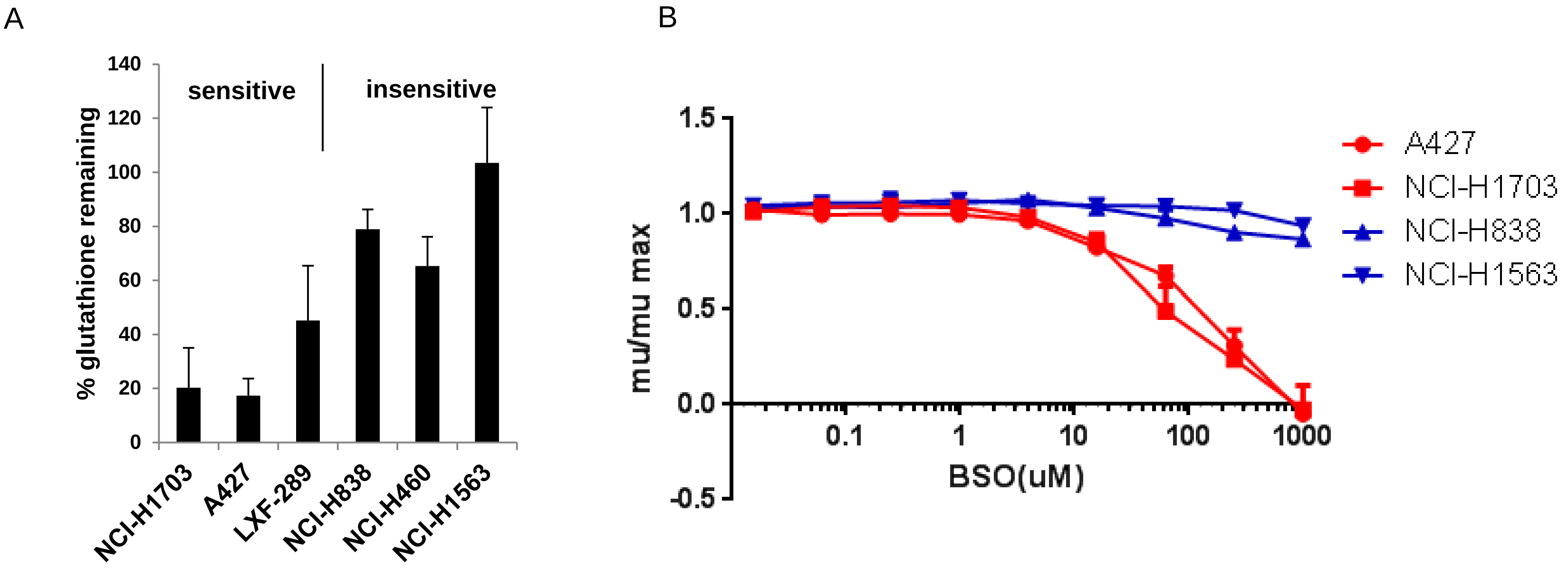

A
% glutathione remaining
140
120
100
80
60
40
20
0
sensitive
insensitive
NCI-H1703
A427
LXF-289
NCI-H838
NCI-H460
NCI-H1563
B
mu/mu max
1.5
1.0
0.5
0.0
-0.5
0.1
1
10
100
1000
BSO(uM)
A427
NCI-H1703
NCI-H838
NCI-H1563

Figure S8

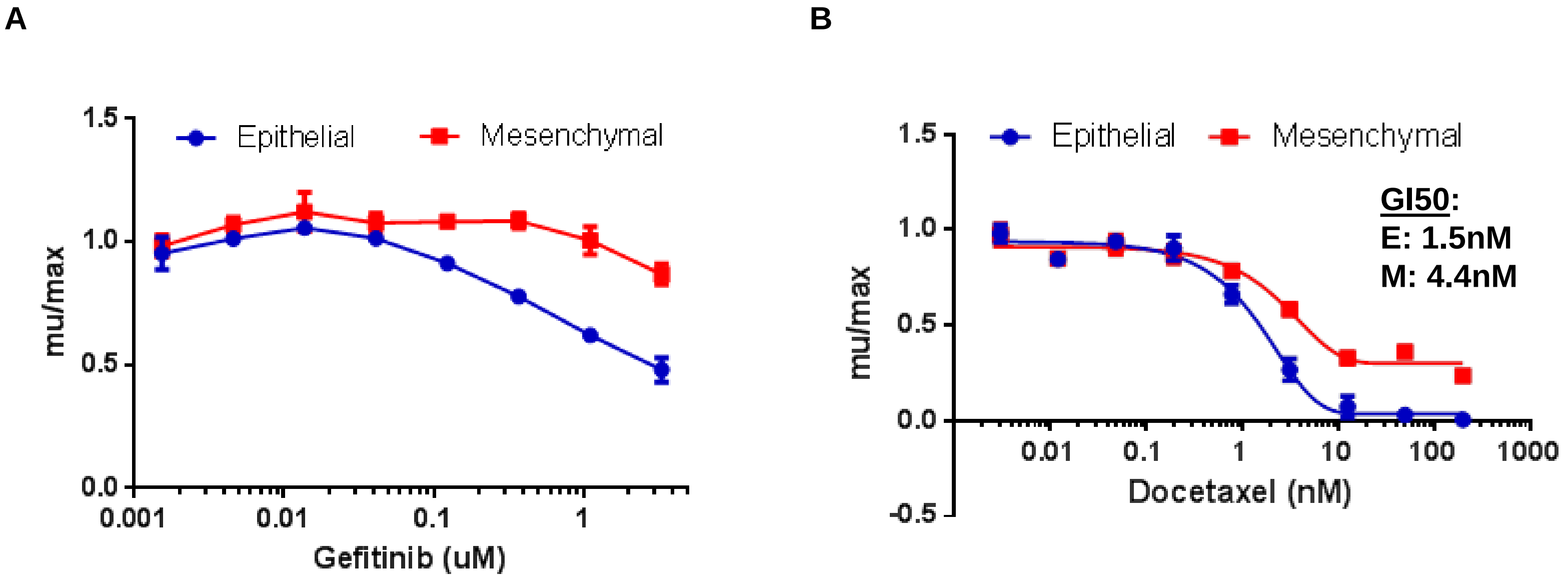
A
B
Epithelial
Mesenchymal
mu/max
1.5
1.0
0.5
0.0
0.001
0.01
0.1
1
Gefitinib (uM)
Epithelial
Mesenchymal
GI50:
E: 1.5nM
M: 4.4nM
mu/max
1.5
1.0
0.5
0.0
-0.5
0.01
0.1
1
10
100
1000
Docetaxel (nM)

Figure S9

Supplemental Figure Legends

Figure S1. Comparison of two different methods of viability measurement. Equivalent A427, NCI-H1703, and NCI-H1563 cells were plated into 96-well plates and treated with DMSO or 8μM BPTES. Growth was measured at the indicated times by (A) CellTiter-Glo (CTG) or (B) Syto60 as indicated. *P* values at 72 hr timepoint comparing growth +/- BPTES by CTG or Syto60: CTG: A427 P=6.9x10^{-9}, NCI-H1703 P=1.4x10^{-9}, NCI-H1563 P=0.42; Syto60: A427 P=1.1x10^{-7}, NCI-H1703 P=1.2x10^{-9}, NCI-H1563 P=0.002.

Figure S2. Diagram highlighting contribution of glutamine and GLS1 activity to the generation of glutamate, aspartate, and TCA intermediates. The five carbons of glutamine highlighted in red represent the fully labeled ^{13}C isotopomer, ^{13}C(5)-glutamine. These ^{13}C atoms can be tracked through the immediate downstream product of glutaminase activity to ^{13}C(5)-glutamate (boxed, right) and after multiple enzymatic steps to ^{13}C(4)-aspartate (boxed, left).

Figure S3. Structures of BPTES and the inactive analogue, AGX-4769.

Figure S4. GLS1 KD and validation of GLS1 antibody in BPTES sensitive vs insensitive NSCLC lines. The indicated NSCLC lines were transfected with either non-targeting siRNAs or siRNAs targeting total GLS1(KGA + GAC) or specifically the GAC isoform. (A) Western blot analysis demonstrating KD of GLS1; the different GLS1 isoforms, KGA and GAC, are indicated. β-actin serves as a loading control. (B) Effects of GLS1 KD on growth as measured by CTG in a 72hr proliferation assay. Results representative of 2 independent experiments.

Figure S5. Quantitation of oxygen consumption rate (OCR). OCR of NCI-H358 epithelial and mesenchymal lines at (A) baseline (P=0.04), and (B) 2 (P=0.34) and 20hrs (P=0.06) post DMSO or 8μM BPTES treatment. (C) Relative cell number as determined by Syto60 staining of cells at the end of the 20hr drug treatment (mean +/-SD).(D) OCR of NCI-H358 epithelial and mesenchymal lines following treatment with increasing concentrations of FCCP. $P=3.8\text{x}10^{-5}$ comparing OCR at 5μM FCCP. Data normalized to OCR at time zero (100%) and presented as mean values +/-SEM.

Figure S6. Diagram highlighting flow of carbon from glucose through glycolysis and one round into the TCA. The six carbons of glucose highlighted in blue represent the fully labeled ^{13}C isotopomer ^{13}C(6)-glucose. Two carbons from glucose get incorporated via Acetyl-CoA into citrate by combining with four carbons from oxaloacetate (OAA).

Figure S7. Graphic summary of metabolic changes induced by BPTES in the epithelial and mesenchymal NCI-H358 cells. Metabolite pool size (depicted by scale of pie circle size) and label distribution (depicted by colored pie pieces) is shown for selected metabolites (black font). Blue and red rectangle borders indicate metabolite labeling from $^{13}C_5$-Gln or $^{13}C_6$-Glc, respectively. $^{13}C_5$-Gln labeling demonstrates effective inhibition of Gln→Glu conversion by BPTES in both lines, though citrate pool sizes are reduced to lower levels selectively in the mesenchymal line. ^{13}C-Glc labeling shows a dramatic reduction in Glc contribution to citrate pools upon BPTES treatment selectively in the mesenchymal line, which is accompanied by a block in flow from DHAP/GA3P to 3-PG (compare pie charts on right for DHAP and 3PG for the two lines).

Figure S8. Decreases in intracellular glutathione levels upon GLS inhibition and dependence on glutathione biosynthesis mark sensitive lines. (A) Intracellular glutathione levels remaining after 24hr treatment with 10μM BPTES in 6 NSCLC lines. Cell lines arranged in order of decreasing sensitivity from left to right (as determined from a 72hr proliferation assay carried out in parallel with the glutathione measurements). Results are plotted as average and standard deviation of 3 independent experiments. (B) The indicated cell lines (BPTES sensitive in red, insensitive in blue) were treated with BSO in a 72hr CTG assay. Results representative of 2 independent experiments and plotted as mean +/-SD.

Figure S9. Decreased sensitivity of mesenchymal NCI-H358 cells to EGFRi and docetaxel compared to epithelial line. NCI-H358 epithelial and mesenchymal lines were treated in triplicate with (A) gefitinib or (B) docetaxel at the indicated concentrations for 72 hrs and cell growth assessed by CTG. Results plotted as average growth rates (+/-SD) compared to DMSO treated cells. The GI50 for docetaxel in the 2 lines is indicated.

Supplemental Methods

GLS1 siRNA knockdown

Cells were transfected with non- targeting pool (5'-UGGUUUACAUGUCGACUAA-3', 5'-UGGUUUACAUGUUGUGUGA-3', 5'-UGGUUUACAUGUUUUCUGA-3', 5'-UGGUUUACAUGUUUUCCUA-3'), GAC (5'-GUAUAAAGGCAGUAGAUUAUU-3'), or KGA/GAC (5'-GCACAGACAUGGUUGGUAU-3') siRNA oligonucleotides (Dharmacon), using Lipofectamine RNAiMAX (Life Technologies) for a final concentration of 20nM. Cells were plated into a 10cm plate at 30-40% confluence and incubated overnight at 37°C, 5% CO_2 in RPMI1640 media containing 10% FBS. 18hrs post plating cells, siRNAs were diluted in OPTIMEM Reduced Serum Media (Life Technologies) and 15ul Lipofectamine RNAiMAX was diluted in 1.5mls in OPTIMEM Reduced Serum Media. siRNA and Lipofectamine mixtures were combined and allowed to incubate at room temperature for 10min (total volume = 2.5ml/10cm plate). Media was removed from cells and washed with PBS. Transfection mixture was added to cells and incubated for 5hrs at 37°C, 5%CO_2. Transfection mixture was removed from cells and replaced with fresh RPMI1640 media +10%FBS. ~40hr post transfection cells were harvested and plated for a 72hr proliferation assay. Protein extracts were harvested 5 days post-transfection.
